# Assessment of Ubiquitous Promoters Driving Fluorescent Marker and Transposase Expression to Develop a High-Performance *piggyBac* Transgenic System in *Bactrocera dorsalis*

**DOI:** 10.3390/insects17030349

**Published:** 2026-03-23

**Authors:** Helin Jiang, Yulun Wu, Jun Cai, Xianwu Lin, Rihui Yan

**Affiliations:** 1 Institute of Tropical Agriculture and Forestry, Hainan University, Danzhou 571700, China; jianghl2026@163.com (H.J.);; 2 Key Laboratory of Green Prevention and Control of Tropical Plant Diseases and Pests, Ministry of Education, Hainan University, Danzhou 571700, China; 3 National Nanfan Research Institute, Chinese Academy of Agricultural Sciences, Sanya 572019, China

**Keywords:** *Bactrocera dorsalis*, *BdActin*, *BdPUb*, hyPBase, transgenic, fluorescent expression

## Abstract

*Bactrocera dorsalis* (oriental fruit fly) is a destructive invasive pest threatening global agriculture. Although integrated pest management is applied, environmentally friendly genetic control methods are urgently needed. The development of such methods particularly relies on efficient genetic elements. In this study, we compared the transient expression of mScarlet-I driven by various *Actin* and *PUb* promoters in *B. dorsalis* embryos. The truncation of two strong promoters, *BdActA3a* and *BdPUb*, revealed that the 5.0-kb *BdActA3a* and 3.6-kb *BdPUb* promoters drove significantly higher expression than their truncated variants. Notably, the *BdPUb* promoter was highly effective in driving fluorescent protein expression in *B. dorsalis*. Using the 3.6-kb *BdPUb* promoter, we constructed a transposase plasmid *BdPUb*-3.6 kb>*hyPBase*. By co-injecting the *BdPUb* 3.6kb>*mScarlet-I* donor construct, we successfully generated a fluorescent transgenic strain with a transgenic efficiency of approximately 26%. The strain exhibited stage-specific fluorescence and maternal effect and the homozygotes showed fecundity comparable to wild-type controls. The high performance of the *piggyBac* transposase and the fluorescence screening system provides a substantial technical foundation for basic research and future development of genetically modified strains to control *B. dorsalis*.

## Simple Summary

This study aimed to identify high-efficiency promoters in *Bactrocera dorsalis*. We compared the ability of different *Actin* and *polyubiquitin-C* (*PUb*) promoters to drive the expression of the fluorescent protein mScarlet-I in *B. dorsalis* embryos. The results showed that the 5.0-kb *BdActA3a* and 3.6-kb *BdPUb* promoters had significantly stronger activity than their truncated variants, and the *BdPUb* promoter showed particularly high performance. Using the 3.6-kb *BdPUb* promoter and *piggyBac*-mediated transgenesis technology, we successfully developed a high-performance *piggyBac*-mediated transgenic system and constructed a fluorescent transgenic strain with a transformation efficiency of approximately 26%. This strain displayed stage-specific fluorescence expression, stable inheritance, and no negative impact on fecundity. Our findings lay a solid foundation for developing genetically modified strains for the genetic control of *B. dorsalis* in the future.

## 1. Introduction

The oriental fruit fly, *Bactrocera dorsalis* (Hendel), a member of the Tephritidae family of Diptera, is one of the most damaging invasive pests. It inflicts substantial economic losses throughout Africa, South America, North America, Asia, and the Pacific Islands by destroying over 250 fruit and vegetable crops [1,2,3,4,5]. Due to its characteristics of broad adaptation, high dispersal capacity, and high reproductive capacity, *B. dorsalis* is now considered as one of the most important pest species in global agriculture [6]. Currently, chemical insecticides are used as the most effective tool to control the populations of *B*. *dorsalis* [7,8]. However, long-term, excessive and inappropriate application of insecticides has led to the widespread development of insecticide resistance in *B*. *dorsalis*, resulting in a continuous decline in control efficacy, which has become a major obstacle in its sustainable management [9,10]. In addition, the extensive use of chemical pesticides leads to pesticide residues in soil, water and agricultural products, posing a threat to environmental safety [11]. It also causes severe harm to important pollinators such as honeybees, affecting the stability of agroecosystems and biodiversity [12]. Pesticide residues can further enter the human body through the food chain, representing a potential risk to human health [13]. Consequently, there is an urgent need to develop more effective and safer methods to control it sustainably.

Multiple environmentally friendly genetic control methods have been developed as alternatives to traditional insecticides for managing tephritid fruit fly pests [14,15,16,17]. The Sterile Insect Technique (SIT), a core strategy of traditional genetic control [18], has been successfully employed in *Ceratitis capitata* and other tephritid pests [19]. However, its extensive application faces several bottlenecks, including difficulties in male-female separation, high costs of mass rearing and release, and reduced mating competitiveness of males caused by radiation treatment [20]. To overcome these disadvantages and develop safe, efficient, and sustainable control strategies for tephritid pests, the exploration of novel molecular technology-based control approaches has become a global research priority.

In recent years, breakthrough progress has been achieved in multiple tephritid species, including *C. capitata*, *B. dorsalis* and *Zeugodacus cucurbitae*, for example, female-specific Release of Insects carrying a Dominant Lethal (fsRIDL), CRISPR/Cas9-mediated gene drive strategies, RNA interference (RNAi)-based biopesticides, natural-source biopesticides, biocontrol microbes and natural enemies, and plant-derived active substances [17,21,22,23,24]. Using CRISPR-based gene editing, researchers have successfully created systems that convert genetic females into fertile or sterile males, leading to male-biased populations and potential population suppression [21,25]. Introducing a specific mutation of the *tsl* gene, which is responsible for a temperature-sensitive lethal phenotype, into a wild-type strain of *C. capitata* via gene editing, resulted in complete embryonic lethality under heat stress [26]. RNA interference (RNAi)-based strategies also represent a promising avenue. Studies have shown that silencing vital genes, such as *E75*, *V-ATPase*, and *RPS13*, significantly reduces male fertility and female fecundity [27,28]. To overcome the instability of dsRNA in the insect gut, researchers have successfully employed nanocarriers (e.g., chitosan and star polycations) and novel circular dsRNA produced in *E. coli* [27,28]. Furthermore, co-suppressing gut nucleases that degrade dsRNA dramatically enhances the RNAi effect, achieving high mortality in adult *C. capitata* [14]. These advances have provided novel approaches for replacing traditional chemical control and achieving species-specific and environmentally friendly management of these pests.

Numerous studies have confirmed that the sex determination process in tephritid fruit flies is mainly regulated by key genes such as *Maleness-on-the-Y* (*MoY*), *tra*, *tra-2*, and *dsx* [29,30,31,32], which provide important molecular targets for the development of novel genetic control strategies, such as fsRIDL. Based on the molecular mechanism of sex determination, the construction of Transgenic Sexing Strains (TSSs) and their mass release to mate with wild pest populations can effectively achieve the suppression or even eradication of wild tephritid populations. These approaches offer species specificity, environmental friendliness, and long-term efficacy and have been successfully applied in various species, including *Drosophila suzukii* [33], *C*. *capitata* [34], *Aedes aegypti* [35], and *Spodoptera litura* [36]. However, the development of TSSs for *B. dorsalis* population control remains rare, largely because adequate genetic research tools are not available.

In genetic engineering, the *piggyBac* transposon system is a widely used molecular tool for inserting DNA into insect genomes [37]. It has been successfully applied for transgenesis in various insect species [20,38]. Fluorescent proteins, such as GFP, DsRed, and mScarlet-I, are commonly used as transgenic markers [39,40]. However, the expression levels and efficacy of these fluorescent proteins depend not only on their intrinsic properties but also significantly on the activity of their respective promoters. A recent study identified an endogenous promoter, *ZcPUb*, capable of driving high-level expression of fluorescent proteins in *Z*. *cucurbitae* [41]. When the highly active piggyBac transposase was placed under the control of this promoter, the transformation efficiency was significantly increased to 12.30% ± 3.17%. This enhanced transposase system was successfully used to rescue puparium color in *wp*^(−)^ individuals by introducing *wp* gene rescue alleles [42]. These results suggest that the use of robust native promoters can substantially increase transgenesis efficiency. In *B. dorsalis*, several studies have successfully established transgenic lines using the *piggyBac* transposon system. Currently, most related studies employ the *Drosophila melanogaster* hsp70 promoter to drive transposase expression; however, the overall transformation efficiency of this system is relatively low, generally only approximately 2% [43,44]. Therefore, a systematic effectiveness evaluation of various promoters in *B. dorsalis* is crucial for advancing genetic control technologies against this pest and facilitating their practical application.

Strong endogenous promoters are key regulatory elements for the efficient and stable expression of target genes, and thus directly determine the screening efficiency and phenotypic stability of transgenic strains. Actin genes are generally constitutively and highly expressed in insect cells, characterized by stable driving efficiency and a broad expression profile [45]. Ubiquitin gene promoters generally exhibit strong promoter activity in eukaryotes and have been confirmed to significantly improve gene expression levels in various insect transgenic systems [46]. Meanwhile, compared with eGFP and DsRed, the novel fluorescent protein mScarlet-I offers multiple advantages, including greater fluorescence brightness, stronger stability, and lower background interference [47], which can significantly improve the efficiency and accuracy of transgenic screening.

In this study, we evaluated the activity of upstream regulatory regions of *Actin cytoplasmic A3a* (*BdActA3a*, LOC105222768) and *polyubiquitin-C* (*BdPUb*, LOC105226185) genes by transiently expressing mScarlet-I in *B. dorsalis*. Considering the promoter length and fluorescent protein expression level, we selected the 3.6-kb *BdPUb* promoter to drive transposase and fluorescent protein expression, and constructed the *BdPUb*-3.6 kb>*hyPBase* expression vector and *BdPUb*-3.6 kb>*mScarlet-I* fluorescent transgenic strains. Verification results showed that this transposase could significantly improve the transposition efficiency.

## 2. Materials and Methods

### 2.1. Insect Rearing

The wild-type (WT) *B*. *dorsalis* were collected from the Haidian Campus of Hainan University and reared in the laboratory under the conditions of 26 °C, 70% relative humidity, 14 h light and 10 h dark per day. Adult flies were provided with water and fed a mixture of sugar and brewer’s yeast powder at a ratio of 3:1, and paper was placed under the food to prevent it from getting damp. The eggs were collected on fresh guava. After hatching, the larvae were fed artificial food, which was developed from the larval diet for *Z*. *cucurbitae* [48] by replacing pumpkin with guava. The third-instar larvae were transferred to boxes containing moist sand to undergo pupation.

### 2.2. Promoter Cloning and Plasmid Construction

Genomic DNA was extracted from *B. dorsalis* adults using the TIANamp Genomic DNA Kit (TIANGEN, Beijing, China). The promoter (including the promoter and potential upstream regulatory elements, such as enhancers, hereinafter referred to as ‘promoter’ for simplicity) sequences of the genes were retrieved from the NCBI database (each sequence contains a 5′-UTR): *BdActin2* (*BdAct2*, LOC105225453); *BdActin5* (*BdAct5*, LOC105228222); *BdActinA3a* (*BdActA3a*, LOC105222768); and *BdPUb* (*polyubiquitin-C*, LOC105226185). Moreover, the promoter regions of *BdPUb* and *BdActA3a* were analyzed using the Neural Network Promoter Prediction (NNPP, version 2.2) (Berkeley Drosophila Genome Project, Berkeley, CA, USA), and a series of 5′-truncated sequences were designed. The target fragments were amplified using 2× PrimeSTAR^®^ Max DNA Polymerase (Takara, Dalian, China). Polymerase chain reaction (PCR) was performed under the following conditions: initial denaturation at 98 °C for 30 s, 35 cycles of 98 °C for 10 s, 55 °C for 15 s, and 72 °C for 5 s/kb, followed by a final extension at 72 °C for 5 m. Primer synthesis and DNA sequencing were conducted by Guangzhou IGE Biotechnology Co., Ltd. (IGE, Guangzhou, China).

PCR products were gel-purified, followed by double digestion with NEB restriction enzymes (NEB, Beijing, China). The pBac: mScarlet-I-SV40 vector fragment in this study was generated using the laboratory plasmid pBac_ZcPUb_mScarlet_sv40 (Accession number: PX262392, NCBI) as the template. The fragment was digested with the corresponding restriction enzymes (NEB) compatible with each promoter, and ligated using T4 DNA Ligase (M0202T, NEB). The corresponding restriction enzyme sites, as well as the NCBI accession numbers and coordinates of the promoters, are listed in Table S1.

The modified transposase plasmid used for *Z. cucurbitae* transgenesis [41] and the 3.6-kb *BdPUb* promoter were digested with *EcoR*I and *Mlu*I (NEB). The digested fragments were gel-purified and then ligated using NEB T4 DNA Ligase to construct the recombinant plasmid *BdPUb*-3.6 kb>*hyPBase*. All recombinant plasmids constructed in this study were transformed into *Escherichia coli* Top10 competent cells (Sangon Biotech, Shanghai, China). The plasmids used in this study are listed in Table S2.

All the primers used for plasmid construction are listed in Table 1. Restriction enzyme sites and protective bases are in bold in the primer sequences.

### 2.3. Embryo Microinjection

The constructed plasmids were extracted using the iPure Rapid Endotoxin-Free Plasmid Mini-Midi Extraction Kit (IGE, Guangzhou, China). Plasmids used for detecting transient fluorescent expression were individually dissolved in injection buffer (5 mM KCl, 0.1 mM Na_3_PO_4_, pH 7.4) at a final concentration of 1000 ng/μL. Fresh eggs were collected within 15 m using guava slices, treated with 1% NaClO for 20 s, then treated with 0.02% Triton X-100 for 20 s, and subsequently rinsed repeatedly with ddH_2_O for 60 s. The treated eggs were quickly arranged on a glass slide using double-sided tape and covered with a 1:1 (*v*/*v*) mixture of Halocarbon 700/27 oil (Sigma, St. Louis, MO, USA). The solution was injected into the posterior end of the embryos at 16 °C using a microinjector FemtoJet 4i (Eppendorf, Hamburg, Germany), an M-152 micromanipulator (NARISHIGE, Tokyo, Japan), and a Leica DM500 microscope (Leica Microsystems, Wetzlar, Germany). After injection, the excess mineral oil was removed, and the slides with embryos were placed in a humidified plastic box at 26 °C.

### 2.4. RNA Extraction, RT-PCR, and RT-qPCR Analysis

Thirty eggs were collected at each developmental stage, and total RNA was extracted using TRI Reagent^®^ (Sigma-Aldrich, St. Louis, MO, USA) following the manufacturer’s instructions. The concentration of the extracted RNA was determined using an ND-100 Ultra-Micro UV-Vis Spectrophotometer (MIULAB, Hangzhou, China). A total of 1 μg of RNA was reversely transcribed into cDNA using NovoScript^®^ Plus All-in-one 1st Strand cDNA Synthesis SuperMix (gDNA Purge) (Novoprotein, Suzhou, China).

RT-PCRs were performed with 2× SapphireAmp^®^ Fast PCR Master Mix (Takara, Dalian, China). The RT-PCR conditions were set as follows: initial denaturation at 95 °C for 1 m, followed by 30 cycles of denaturation at 95 °C for 5 s, annealing at 56 °C for 5 s, and extension at 72 °C for 5 s. A final extension step was performed at 72 °C for 5 m. *α-tubulin* was used as the reference gene. RT-qPCR assays were conducted on an AMolarray MA-6000 Real-Time Fluorescent Quantitative PCR System with NovoStart^®^ Universal Fast SYBR qPCR SuperMix (Novoprotein, Suzhou, China), and three independent biological replicates were performed for each sample. *α-tubulin* was employed as the internal reference for data normalization [49]. All the primers used in this study are listed in Table 1.

### 2.5. Fluorescence Observation and Image Processing

Fluorescence in the embryos was observed 15 h post-injection using a SZX16 stereomicroscope (Olympus, Tokyo, Japan) and photographed with a DP72 digital camera (Olympus, Tokyo, Japan). The expression of mScarlet-I was detected using an RFP filter (Ex540–580 nm, Em > 610 nm). All images were processed using Adobe Photoshop (2025) for brightness and contrast adjustment. Quantitative analysis of embryonic fluorescence was performed using ImageJ 1.54f (National Institutes of Health, Bethesda, MD, USA), and the data were normalized according to the relative fluorescence intensity. All bar charts were plotted using GraphPad Prism 9.0 (GraphPad Software, San Diego, CA, USA).

### 2.6. Development of Fluorescent Strains

To generate transgenic fluorescent strains, a mixture of the plasmid expressing the fluorescent protein mScarlet-I (500 ng/μL) and the *BdPUb*-3.6 kb>*hyPBase* plasmid (500 ng/μL) was prepared in injection buffer. The mixture was microinjected into embryos. G0 fluorescent adults were selected and subjected to single-pair (one male × one female) mating with WT adults of the opposite sex. The laid eggs were collected, and fluorescent G1 transgenic individuals were screened. After G1 adults emerged, they were mated with WT adults of the opposite sex in single pairs, and G2 transgenic individuals were collected. Upon their emergence, G2 siblings were crossed with each other to obtain homozygous transgenic individuals.

### 2.7. Identification of Transgenic Elements and Insertion Sites

Genomic DNA (gDNA) was extracted from the single middle leg of transgenic fluorescent G2 adults using the TIANamp Genomic DNA Kit (TIANGEN, Beijing, China). The integration site was identified by the Splinkerette PCR (Splink PCR) method [50]. The PCR products containing the genomic sequence flanking the 3′ end of *piggyBac* were then sent for sequencing. The primers used for Splink PCR are listed in Table 1. Homozygous mutants were screened by PCR using the 15-F and 15-R primers (Table 1). When the transgene was successfully integrated, a band of 5933 bp was detected.

### 2.8. Fertility Test

After the identified homozygous fluorescent strains were maintained for five generations, 10 newly emerged females and 10 males were randomly selected and crossed in the same cage, with three biological replicates set up. WT adults were used as controls. Eggs were collected from each cross for 10 consecutive days between 12:00 and 16:00 starting from day 15. The numbers of eggs and hatched larvae were recorded, and the hatching rates were calculated.

## 3. Results

### 3.1. The BdActA3a Promoter Induces Significantly Higher Fluorescence Expression than BdAct2 and BdAct5 in B. dorsalis

**Figure 1:**
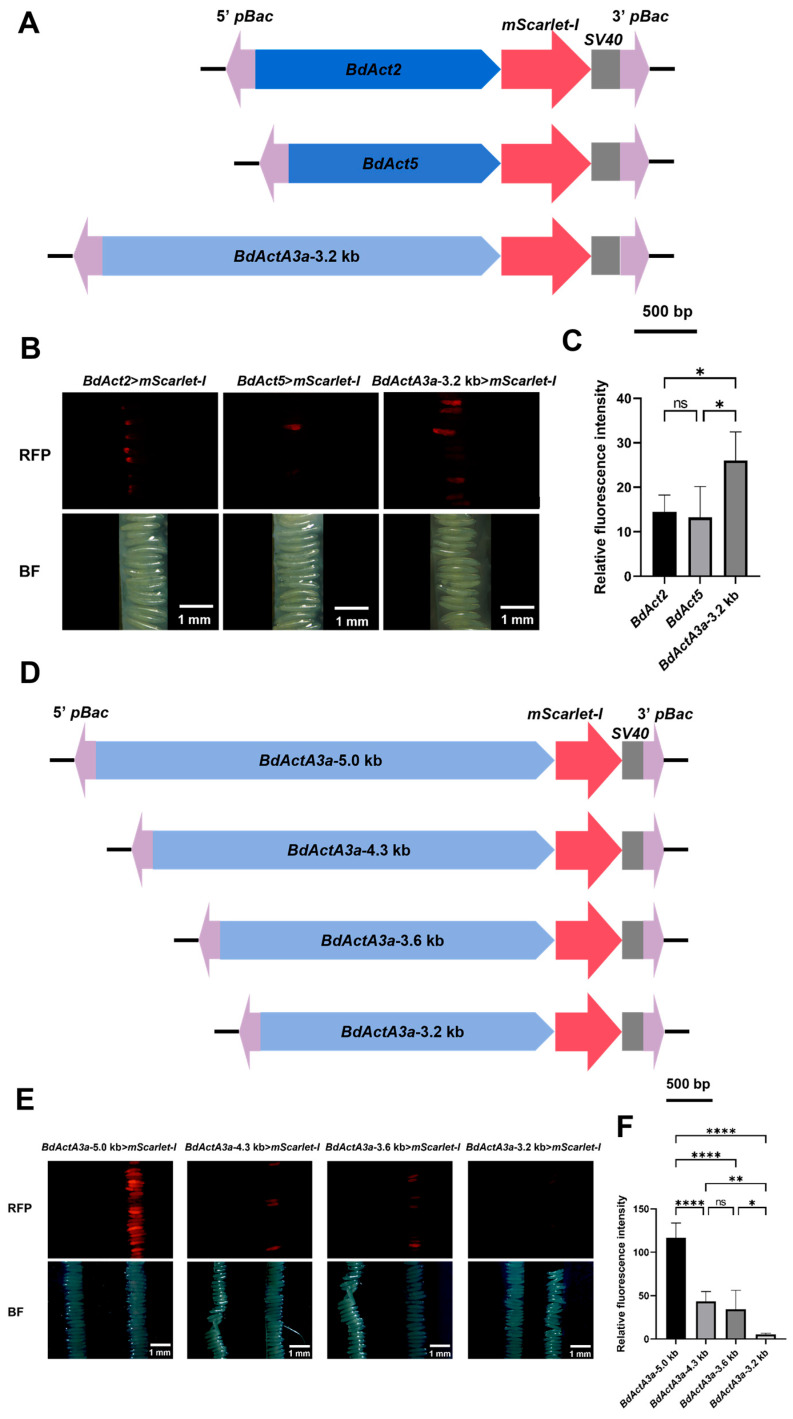
Fluorescent expression regulated by different *Actin* promoters in *B. dorsalis.* (**A**) Schematic diagram of constructs for transient expression of mScarlet-I driven by different *BdActin* promoters in *B. dorsalis*. The promoter lengths of *BdAct2*, *BdAct5*, and *BdActA3a* are 1960 bp, 1690 bp, and 3180 bp, respectively. (**B**) Fluorescence microscopy images of embryos 15 h post-injection with *BdAct2*>*mScarlet-I*, *BdAct5*>*mScarlet-I*, and *BdActA3a*-3.2 kb>*mScarlet-I*. The upper panels show the embryos observed under excitation light, and the lower panels show the embryos observed under bright field. Imaging parameters: exposure time of 100 ms, ISO 400. Scale bars: 500 μm. (**C**) Analysis of fluorescence intensity driven by different *BdActin* promoters. Quantitative analysis of the fluorescence images was performed using ImageJ. A one-way analysis of variance (ANOVA) was used for verification (*p* < 0.05). (**D**) Schematic diagram of constructs for transient expression of mScarlet-I driven by *BdActA3a* promoters of varying lengths. The *BdActA3a* promoter (4958 bp) was truncated to 4349 bp (*BdActA3a*-4.3 kb promoter), 3622 bp (*BdActA3a*-3.6 kb promoter), and 3180 bp (*BdActA3a*-3.2 kb promoter). (**E**) Fluorescence microscopy images of embryos injected with *BdActA3a* transient expression plasmids of different lengths. The upper panels show the embryos observed under excitation light (RFP), while the lower panels show the embryos observed under bright field (BF). In each panel, the left side represents embryos from the injection treatment group, while the right side represents WT embryos. Imaging parameters: exposure time of 10 ms, ISO 400. Scale bars: 500 μm. (**F**) Analysis of fluorescence intensity driven by *ActA3a* promoters of different lengths. Quantitative analysis of the fluorescence images was performed using ImageJ. A one-way analysis of variance (ANOVA) was used for verification (*p* < 0.05). *, **, ****, and ns denote *p* < 0.05, *p* < 0.01, *p* < 0.0001, and *p* > 0.05, respectively.

We first systematically evaluated the promoter activities of *Actin* genes: *BdActin2* (*BdAct2*, LOC105225453), *BdActin5* (*BdAct5*, LOC105228222), and *BdActinA3a* (*BdActA3a*, LOC105222768) (Figure 1A). The promoters were used to drive the expression of mScarlet-I, which is a truly monomeric red fluorescent protein with superior cellular performance including low cytotoxicity, rapid and efficient chromophore maturation, and excellent intracellular brightness [40]. Since two transcripts of the *BdActA3a* gene were predicted, and the long variant contains an intron of 2983 bp in its 5′ UTR (Figure S1), we first obtained a fragment of 3.2-kb by extending approximately 2.3-kb upstream from the short transcript. This fragment was then used as a promoter of *BdActA3a* to drive mScarlet-I expression. Different transient expression plasmids of *BdActin* were injected into *B*. *dorsalis* embryos (Table S3). Fluorescence expression was observed at 15 h post-microinjection with an exposure time of 100 ms. Different promoters showed distinct fluorescence expression patterns. Embryos injected with *BdAct2*>*mScarlet-I* exhibited minimal fluorescence expression restricted to the posterior end of the embryos, with a relative fluorescence intensity (RFI) of 14.46. *BdAct5*>*mScarlet-I* showed negligible fluorescence with a RFI of 13.22, whereas *BdActA3a*-3.2 kb>*mScarlet-I* displayed strong red fluorescent signals globally with a RFI of 24.02 (Figure 1B,C).

When the 3.2-kb promoter of *BdActA3a* (based on the short transcript) was used, it only induced strong red fluorescence at an exposure time of 100 ms. Based on this observation, we further obtained a sequence of approximately 5.0 kb by extending 1.8 kb upstream from the long transcript. We then truncated this 5.0-kb sequence to generate fragments of 4.3 kb, 3.6 kb, and 3.2 kb (the same length we used initially), aiming to identify the key regulatory regions of the *BdActA3a* promoter. Similar transient expression plasmids were constructed for microinjection (Figure 1D). The promoter activity was analyzed by comparing the intensity of transient expression of mScarlet-I. At 15 h after microinjection, fluorescence expression was observed with an exposure time of 10 ms. The exposure time was adjusted from 100 ms to 10 ms for optimal comparison, as some embryos exhibited very strong fluorescence that resulted in overexposure at 100 ms. The results of microinjection for transient expression plasmids with different lengths of the *BdActA3a* promoter are shown in Table S4. The transient fluorescent expression in embryos significantly decreased when the upstream sequence of *BdActA3a* was truncated from approximately 5.0 kb to 4.3 kb, with the RFI decreasing from 116.38 to 43.59, indicating that a regulatory factor exists in this region. When truncated from 4.3 kb to 3.6 kb and then to 3.2 kb, the transient fluorescent intensity in embryos gradually decreased, and the RFI dropped to 34.19 and 5.15, respectively (Figure 1E,F), suggesting that important regulatory elements are also present in this region. These results indicate that the approximately 5.0-kb upstream sequence of *BdActA3a* has better performance than its truncations and those of *Actin* counterpart promoters and can be used to drive fluorescent protein expression in transgenesis of *B. dorsalis*.

### 3.2. Comparison of BdPUb Promoter and Its Truncated Variants in Driving Fluorescence Expression in B. dorsalis

**Figure 2:**
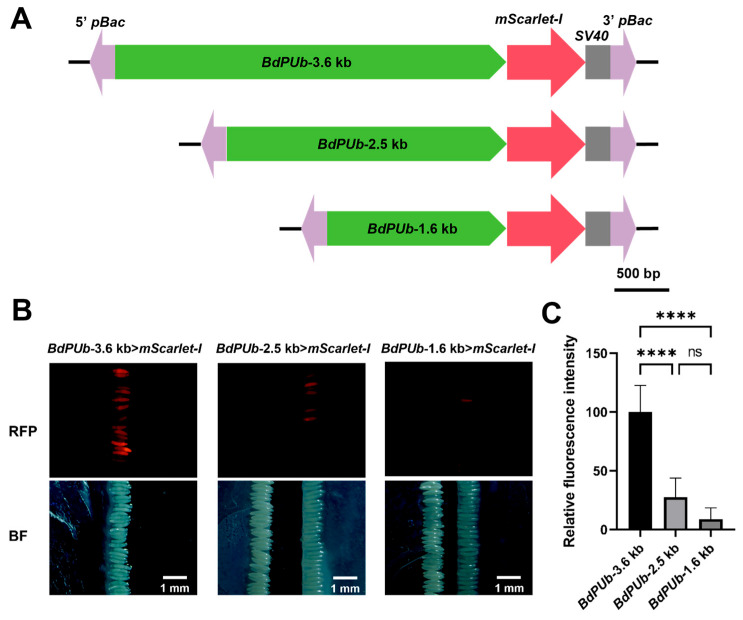
Fluorescent expression regulated by *BdPUb* promoters of different lengths in *B. dorsalis*. (**A**) Schematic diagram of constructs for transient expression of mScarlet-I driven by *BdPUb* promoters of different lengths in *B. dorsalis*. The *BdPUb* promoter (3569 bp) was truncated to 2556 bp (*BdPUb*-2.5 kb) and 1637 bp (*BdPUb*-1.6 kb). (**B**) Fluorescence microscopy images of embryos injected with transient expression plasmids containing *BdPUb* promoters of different lengths. The upper panels show the embryos observed under excitation light, while the lower panels show the embryos observed under bright field. In each panel, the left side represents embryos from the injection treatment group, while the right side represents WT embryos. Imaging parameters: exposure time of 10 ms, ISO 400. Scale bars: 500 μm. (**C**) Analysis of fluorescence intensity driven by *BdPUb* promoters of different lengths. Quantitative analysis of the fluorescence images was performed using ImageJ. A one-way analysis of variance (ANOVA) was used for verification (*p* < 0.05). **** and ns denote *p* < 0.0001 and *p* > 0.05, respectively.

Simultaneously, we assessed the promoter activity of *BdPUb*, as a previous study indicated that the native *PUb* promoter facilitates substantial protein expression within its host organism [41]. We amplified the 3.6-kb upstream sequence of the *BdPUb* gene and created a transient expression plasmid driving mScarlet-I expression. An intense red fluorescence was observed in *B*. *dorsalis* embryos at 15 h post-microinjection, with an exposure time of only 10 ms. To further explore the activity of the *BdPUb* promoter, we truncated the 3.6-kb upstream regulatory sequence to 2.5 kb and 1.6 kb, and constructed two additional transient expression plasmids (Figure 2A). The constructs were microinjected into *B. dorsalis* embryos, and the promoter activities of these different constructs were evaluated by comparing their red fluorescence intensities. The microinjection results are shown in Table S4. The results revealed that truncating the upstream sequence of *BdPUb* from 3.6 kb to 2.5 kb resulted in a significant reduction in the intensity of transient fluorescence expression in embryos, with the RFI dropping from 100.21 to 27.57. Further truncation from 2.5 kb to 1.6 kb caused an even more pronounced decline in fluorescence intensity in embryos, with a drastic drop of RFI to 8.79 (Figure 2B,C). The fluorescence intensity driven by the 5.0-kb *BdActA3a* promoter was similar to that driven by the 3.6-kb *BdPUb* promoter. However, the 5.0-kb *BdActA3a* promoter was too long, carried the risk of promoter silencing, and would increase the metabolic burden. Therefore, we selected the 3.6-kb *BdPUb* promoter for constructing the fluorescent line.

### 3.3. Expression Pattern of mScarlet-I Driven by BdPUb Across Different Developmental Stages of Transgenic B. dorsalis

**Figure 3:**
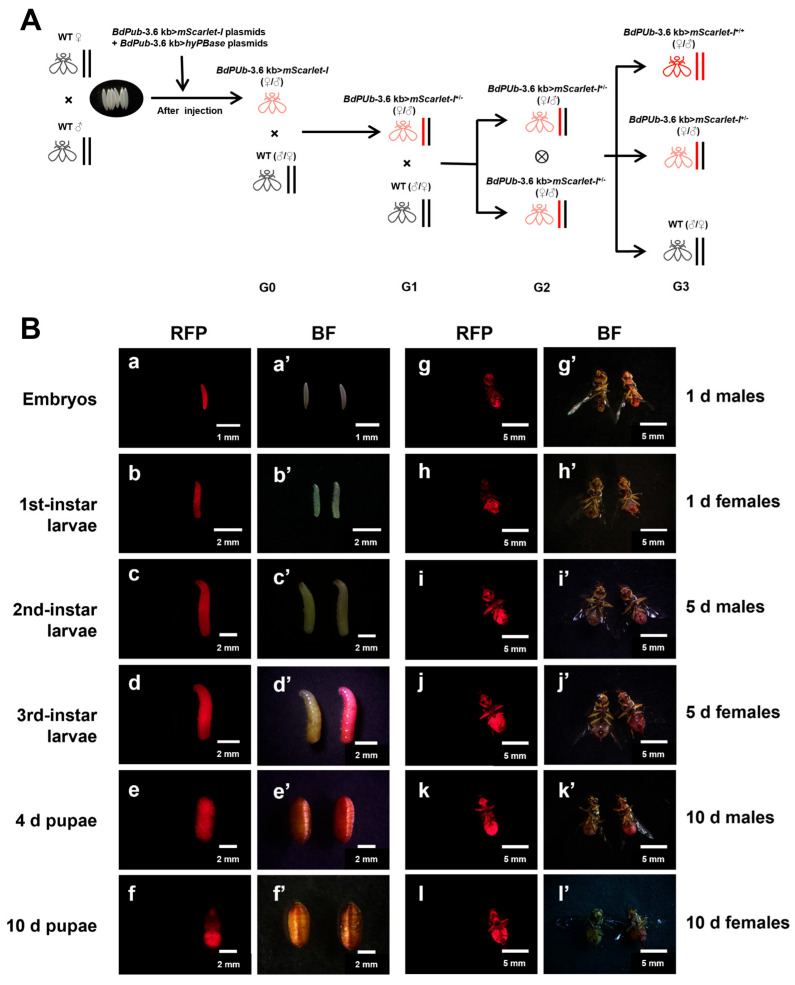
Cross schematic for generating fluorescent expression strains and mScarlet-I expression profiles at different developmental stages of *B. dorsalis*. (**A**) Cross schematic diagram for generating fluorescent expression strains. (**B**) Images of G1 individuals with mScarlet-I fluorescent expression at different developmental stages, including RFP images and BF images. For each image, the fluorescent heterozygote is shown on the right, while the WT counterpart for comparison is shown on the left. Imaging parameters: exposure time of 10 ms, ISO 400. Scale bars: (**a**,**a’**),1 mm; (**b**,**b’**–**f,f’**), 2 mm; (**g**,**g’**–**l**,**l’**), 5 mm.

In prior studies conducted in our laboratory, seven amino acid substitutions were introduced into the catalytic domain of the canonical transposase, which was then combined with the 1.7-kb *ZcPUb* promoter to regulate its expression. This resulted in the creation of a highly active modified hyPBase that demonstrated a significant enhancement in transformation efficiency without exhibiting cellular toxicity [41]. Based on this, we developed a modified transposase that is regulated by the 3.6-kb *BdPUb* promoter, which exhibits moderate mScarlet-I expression activity in *B*. *dorsalis*. The *BdPUb*-3.6 kb>*mScarlet-I* plasmid was mixed with the *BdPUb*-3.6 kb>*hyPBase* plasmid at a 1:1 ratio and injected into *B. dorsalis* embryos. A total of 1329 embryos were injected, yielding 63 viable G0 fluorescent adults (Table S5). Given that the analogous system employed in our laboratory has demonstrated a transformation efficiency of approximately 12% for *Z. cucurbitae* [41], we anticipated a comparable high transformation efficiency for *B. dorsalis*. Nineteen G0 adults were then randomly selected and mated in single pairs with wild-type (WT) adults of the opposite sex to collect G1 eggs. Embryonic fluorescence was examined at 15 h post-oviposition (Figure 3A). Five out of the 19 cages produced fluorescent G1 transgenic embryos (Figure 3(a,a’)), corresponding to a germline transformation efficiency of 26.32%. The numbers of fluorescent G1 individuals obtained from these five positive cages are listed in Table S6. This transformation efficiency is higher than 12.30% germline transformation efficiency observed with *ZcPUb*>*hyPBase* in *Z. cucurbitae* [41], probably due to the elevated hyPBase expression in *B. dorsalis* embryos induced by the 3.6-kb *BdPUb* promoter.

The red fluorescence expression profiles of these individuals were monitored across different developmental stages. We found that from the embryonic stage to the early pupal stage (Figure 3(a,a’–e,e’)), *BdPUb*-3.6 kb>*mScarlet-I* transgenic individuals exhibited ubiquitous red fluorescence throughout the body, with relatively uniform signal intensity and no obvious tissue specificity. However, during the late pupal stage (Figure 3(f/f’)), the red fluorescence of *BdPUb*-3.6 kb>*mScarlet-I* gradually intensified in the abdomen but weakened in the thoracic and head regions, forming distinct non-fluorescent areas around the eyes. In adult individuals (Figure 3(g,g’–l, l’)), red fluorescence was predominantly concentrated in the abdomen, with weaker fluorescence in other regions of the body. In addition, due to the accumulation of mScarlet-I, transgenic larvae and pupae exhibited a slightly redder appearance compared to WT, and the abdomen of transgenic adults also showed a red hue (Figure 3B). This reddish appearance is probably due to the excessive fluorescence, which becomes visible through the cuticle.

### 3.4. Genetic Characteristics and Expression Regulation of BdPUb Transgenic Strains

**Figure 4:**
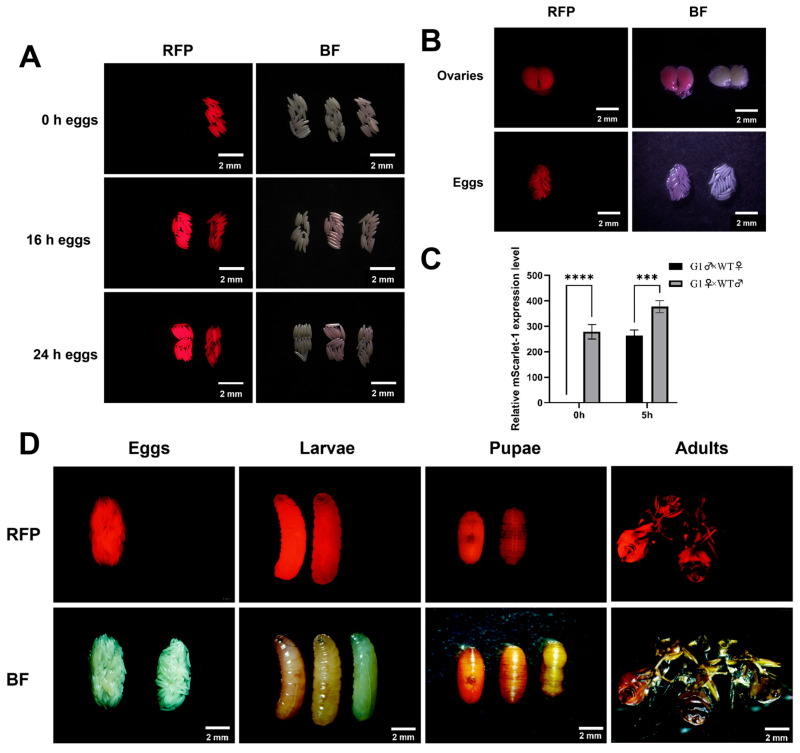
Expression patterns of *BdPUb*-3.6 kb>*mScarlet-I* in transgenic *B. dorsalis*. (**A**) Fluorescence comparison of G2 eggs at 0 h, 16 h and 24 h post-oviposition from crosses of G1 female/male heterozygotes with wild-type flies. The left panels display fluorescent images, while the right panels show bright-field images. For each panel, the leftmost one represents WT eggs; the middle one represents G2 eggs from crosses between G1 males and WT females; and the rightmost sample represents G2 fluorescent eggs derived from crosses between G1 females and WT males. Imaging parameters: exposure time of 10 ms, ISO 400. Scale bars: 2 mm. (**B**) Anatomical images of ovaries in G1 heterozygous females mated with wild-type. The images include dissected intact ovaries and eggs retrieved from the ovaries. The left panels show fluorescent images, while the right panels display bright-field images. WT controls are shown on the right side of each panel for comparison. Imaging parameters: exposure time of 10 ms, ISO 400. Scale bars: 2 mm. (**C**) RT-qPCR statistics graph of mScarlet-I expression dynamics in G2 eggs at 0 h and 5 h post-oviposition. Data are presented as means ± SEM (*n* = 3) and normalized to the transcript levels of *α-tubulin*. Asterisks indicate significant differences determined by *t*-test (*** *p* <0.001, **** *p* < 0.0001). (**D**) Comparative visualization of mScarlet-I fluorescence in transgenic G3 individuals and WT controls. The upper row shows fluorescence images and the lower row shows bright-field images. The leftmost panel displays the comparison between G3 fluorescent eggs and WT eggs; for the remaining panels, G3 individuals are shown in orange-red, pale orange, and yellowish-white (WT) from left to right. Imaging parameters: exposure time 10 ms, ISO 400. Scale bars: 1 mm.

To obtain transgenic strains with a single insertion locus, single-pair crosses were performed between *BdPUb*-3.6 kb>*mScarlet-I* transgenic G1 adults (heterozygotes) and WT individuals. During the collection of G2 eggs, we noticed distinct fluorescence patterns associated with different parental cross schemes. When G1 males were crossed with WT females, none of G2 eggs exhibited fluorescence at 0 h post-oviposition. However, at 16 h post-oviposition, approximately 50% of G2 eggs displayed bright red fluorescence, consistent with the Mendelian segregation ratio expected for a test cross (Figure 4A). In contrast, when G1 females were crossed with WT males, all G2 eggs showed bright red fluorescence at 0 h post-oviposition. The fluorescence intensity of a subset of G2 eggs derived from G1 females showed a slight decrease at 16 h post-oviposition and a more pronounced reduction at 24 h post-oviposition (Figure 4A). Dissection of G1 females revealed that immature eggs within the ovaries already exhibited strong fluorescence signals (Figure 4B), confirming the robust maternal effect of *BdPUb* expression.

RT-qPCR was performed to quantify the expression dynamics of mScarlet-I in G2 embryos at 0 and 5 h post-oviposition, derived from the two cross combinations (G1 male × WT female and G1 female × WT male) (Figure 4C). No mScarlet-I expression was detected in freshly laid eggs from the G1 male × WT female cross. In contrast, freshly laid eggs from the G1 female × WT male cross exhibited high-level mScarlet-I expression (RFI = 278.5). At 5 h post-oviposition, mScarlet-I expression in eggs from the G1 male × WT female cross increased to a relative expression level of 263.8. By comparison, the mScarlet-I expression level in eggs from the G1 female × WT male cross showed a relatively modest increase, rising by approximately 35.97% compared with the level at 0 h post-oviposition.

After conducting the sibling mating of the G2 generation, we observed three distinct body colors in G3 third-instar larvae: yellowish-white, pale orange, and orange-red. We speculated that when the transgenic strains become homozygous, the expression levels of the fluorescent protein increase. Accordingly, homozygous larvae exhibited a visually deeper orange-red color, while heterozygotes showed a pale orange color. Based on this phenotypic correlation, larvae from each color group were reared separately to adulthood. Consistent with larval color phenotypes, the abdominal fluorescence of adults showed the same pattern of zygosity-dependent variation (Figure 4D).

### 3.5. Acquisition and Fecundity Assessment of Homozygous Fluorescent Transgenic Strains of B. dorsalis

**Figure 5:**
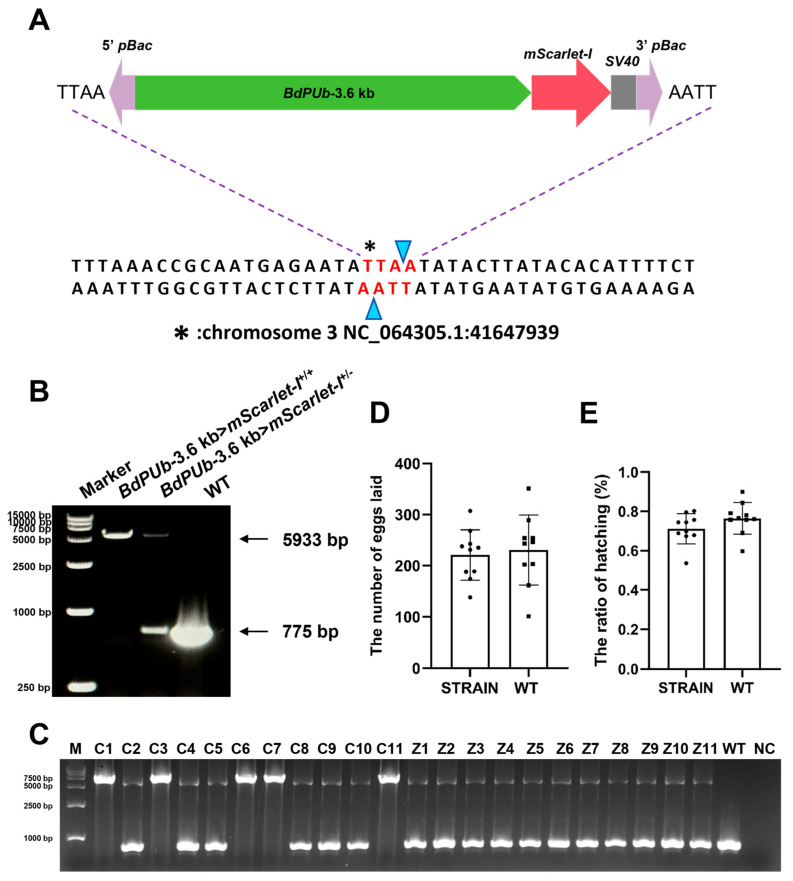
Localization and validation of transgene insertion and evaluation of reproductive fitness of a fluorescent *B. dorsalis* strain. (**A**) Schematic diagram of the transposon insertion site in the fluorescent strain. The insertion site is located at position 41,647,939 on chromosome 3 (NC_064305.1). (**B**) PCR verification for the fluorescent *B. dorsalis* strain. Primers were designed to flank the insertion site. Homozygotes showed a single band of 5933 bp; heterozygotes showed two bands of 5933 bp and 775 bp; WT individuals showed only a single band of 775 bp. (**C**) PCR-based genotyping of third-instar larvae from the transgenic G3 generation. (**D**) Daily egg production of the homozygous fluorescent strain and WT *B. dorsalis*. The *t*-test was used to test the difference (*p* < 0.05). (**E**) Egg hatching rate of the homozygous fluorescent strain and WT *B. dorsalis*. The *t*-test was used to test the difference (*p* < 0.05).

Splink PCR was performed on G2 adults of the transgenic *B. dorsalis* strain to identify the insertion site. The results revealed that the insertion site of this transgenic strain is located in the non-coding region of chromosome 3 (NC_064305.1), starting at position 41,647,939 (Figure 5A). Based on this locus-specific information, we developed a molecular analysis method to distinguish homozygotes and heterozygotes. Homozygous individuals yielded a single band of 5933 bp, whereas heterozygous individuals produced two bands of 5933 bp and 775 bp (Figure 5B), corresponding to the transgenic and WT alleles, respectively. A homozygous *BdPUb*-3.6 kb>*mScarlet-I* transgenic strain was subsequently established through sibling mating of homozygous individuals.

To assess whether the body color of G3 third-instar larvae could serve as a reliable visual marker for homozygosity screening, we selected 11 orange-red larvae (C1–C11) and 11 pale orange larvae (Z1–Z11) for molecular validation. Genotyping by PCR showed that five out of the 11 orange-red larvae were homozygous, whereas all 11 pale orange larvae were heterozygous (Figure 5C). These results indicate that body color alone can reliably indicate heterozygosity but is insufficient to distinguish heterozygotes from homozygotes in the *BdPUb*-3.6 kb>*mScarlet-I* transgenic strains.

To assess the influence of the homozygous fluorescent strain of *B. dorsalis* on reproductive fitness, we conducted a fecundity assay using WT *B. dorsalis* as a control. The statistical analysis showed that there were no significant differences between the homozygous fluorescent strain and the WT strain in daily egg production and larval hatching rate (Figure 5D,E). These results indicate that the insertion of the fluorescent protein expression system into *B. dorsalis* has no impact on its reproduction.

## 4. Discussion

The expression intensity and spatiotemporal pattern of a gene are largely determined by its promoter and other upstream regulatory elements, and the differences in activity and expression patterns among different promoters provide flexible technical support for gene function validation and transgenic tool development. At present, numerous studies have focused on optimizing species-specific endogenous promoters for gene editing research. Researchers have conducted in-depth studies on the *CcAct5C*, *Ccvasa* [25], and *wp* gene promoters of *C*. *capitata* [51]; the *ZcU6* promoter of *Z*. *cucurbitae* [42]; the *Dsvasa* promoter of *D*. *suzukii* [52]; and the testis-specific *Aswampa* promoter of *Anastrepha suspensa* [53]. The core functional regions of these promoters have been successfully identified and are applied in pest genetic control research. However, systematic studies on the endogenous promoters of *B*. *dorsalis* are still relatively scarce. Therefore, this study conducted a series of investigations on the endogenous promoters of *B*. *dorsalis*, aiming to screen for highly active promoters, improve transgenic expression efficiency, and lay a solid foundation for its subsequent genetic control research.

Among these, the promoters of *Actin* and *PUb* genes have considerable advantages due to their involvement in regulating housekeeping gene expression [54,55]. These two types of genes are involved in maintaining basic life activities of organisms, such as cell structure stability and protein metabolism. They theoretically exhibit broad-spectrum and efficient expression in various tissues and at different developmental stages of insects [56,57], making them ideal candidates for driving the continuous expression of exogenous genes.

In this study, we compared the expression intensity of the monomeric red fluorescent protein mScarlet-I driven by different *Actin* promoters and found that the *BdActA3a* promoter yielded stronger fluorescence signals compared to other *Actin* promoters. To further explore its crucial regulatory domains, we then examined the *BdActA3a* promoters of different lengths. When the upstream sequence of *BdActA3a* was truncated from approximately 5.0 kb to 4.3 kb, 3.6 kb, and then 3.2 kb, the transient fluorescent intensity in embryos gradually decreased, indicating that key elements regulating downstream gene expression reside in all three intervals and that the activity of the *BdActA3a* promoter depends on the synergistic contributions from these multiple regions. Notably, truncations from 5.0 kb to 4.3 kb and from 3.6 kb to 3.2 kb led to RFI reductions by 1.67-fold and 5.64-fold, respectively, highlighting the functional importance of these two regions. As a highly conserved structural protein, *BdActA3a* is involved in numerous essential cellular processes. Previous studies have shown that *Actin*-related genes in *B*. *dorsalis* are highly expressed from the egg to pupal stages [58,59], leading us to hypothesize that the *BdActA3a* promoter may also possess broad and robust transcriptional activity. Our transient expression assays confirmed that this promoter can serve as an excellent driver for fluorescent protein expression in *B*. *dorsalis*.

Ubiquitin is a small protein widely present in eukaryotes, primarily known for tagging proteins for degradation via the 26 S proteasome [60]. In addition to protein turnover, ubiquitin also plays a key regulatory role in cell proliferation, differentiation, and stress responses. Moreover, upregulation of *polyubiquitin* genes can enhance the adaptability of organisms to environmental stimuli such as oxidation, heat shock, and proteotoxic stress [57,61]. In *D*. *melanogaster*, the homologous gene *ubi-63E* (*ubiquitin-63E*, Gene ID: FBgn0003943) maintains high expression levels throughout all growth and developmental stages, with significantly elevated expression in late embryos and pupae [62]. Moreover, transgenic *B*. *dorsalis* strains have been successfully generated using the *D. polyubiquitin* promoter to drive DsRed, and the reporter expression was observed across all developmental stages [63]. However, as the homologous gene of *ubi-63E* in *D*. *melanogaster*, the specific expression pattern of the *BdPUb* gene in *B*. *dorsalis* remains unclear at present. In this study, we conducted a functional analysis of the *polyubiquitin* promoter (*BdPUb*) in *B*. *dorsalis*. Embryonic microinjection experiments showed that, at an exposure time of 10 ms and ISO 400, the *BdPUb* promoter drove downstream gene expression with significantly higher efficiency than the *BdActA3a* promoter. Further truncation experiments revealed that the two regions (3.6 kb–2.5 kb and 2.5 kb–1.6 kb) within the upstream sequence of *BdPUb* caused a remarkable decrease in RFI by 72.49% and 68.04%, respectively. This indicates that key elements regulating downstream gene expression exist in both regions and that the activity regulation of this promoter relies on the synergy of multiple regions. This highlights the necessity and scientific value of this investigation into the functions of the *BdPUb* promoter.

More importantly, the transposase *BdPUb*-3.6 kb>*hyPBase* independently constructed in this study significantly improved transposition efficiency. When both the *BdPUb*-3.6 kb>*hyPBase* plasmid and donor plasmid were co-injected at a concentration of 500 ng/μL each, five transgenic G1 individuals were obtained from 19 G0 adults, achieving a transposition efficiency of 26.32%. This represents increases of 1.67-fold and 13.77-fold over efficiencies achieved using transposase mRNA [58] and the transposase under the regulation of an *hsp70* promoter [43], respectively. Our transposase plasmid can be co-injected with any desired plasmid of interest to generate transgenic *B. dorsalis* strains with high efficiency. It should be noted that only 19 randomly selected fluorescent G0 individuals were used for crossing and identification in this study, therefore not all G0 individuals (including both fluorescent and non-fluorescent individuals) were systematically examined. Given that the non-fluorescent G0 adults (i.e., those lacking somatic fluorescent expression) may have transgene insertion in their germline cells, and that the fluorescent phenotype in G0 individuals does not strictly correlate with transgene integration events in the germline, the transposition efficiency reported here only provide a rough estimate of the transformation efficiency within the detectable range and does not accurately represent the overall transgenesis rate. Nonetheless, our data indicate a high transgenesis efficiency when using the endogenous *BdPUb* promoter to control hyPBase expression, thereby promoting the efficient generation of transgenic *B. dorsalis* strains in future studies.

Interestingly, the transgenic larvae expressed fluorescence and developed a pale orange body color. This visible marker enables preliminary identification of transgenic *B. dorsalis* without the need for any equipment. Additionally, homozygous individuals exhibited deeper body color at the third-instar larval stage, allowing preliminary homozygote screening based on color intensity. This method greatly facilitates the follow-up screening process. Furthermore, we observed that the fecundity of transgenic homozygotes was almost identical to that of WT individuals, consistent with the previous report that the *PUb* promoter imposes minimal fitness costs across all developmental stages in transgenic mosquitoes [64]. These findings further support the feasibility of using *BdPUb*-3.6 kb>*mScarlet-I* as a visible genetic marker in *B. dorsalis*.

Pest genetic control strategies, considered as pest population management technologies beneficial to human health, food safety, and sustainable agricultural development, have received extensive attention worldwide since the 1950s [65]. The Sterile Insect Technique (SIT), which involves mass rearing, sterilization, and release of insects to suppress, contain, prevent (re)introduction, or even locally eradicate pest and vector populations [66], has been successfully integrated into Area-Wide Integrated Pest Management (AW-IPM) programs [67]. As a major agricultural fruit-boring pest, *B. dorsalis* demands effective management measures to control its severe damages on crops. This study identified promoters for heritable fluorescent screening markers, provided a reliable screening tool for future studies on gene function, and developed a high-performance *piggyBac*-mediated transgenic system in *B. dorsalis*, therefore laying a solid foundation for its genetic control.

## Figures and Tables

**Table 1:** Primers used in this study.

Name	Primer Sequence (5′→3′)	Purpose
Actin2-F	**CGCGGATCC**CTTGCGCTCCATGTACAAGTGTG	Amplify the promoter sequences
Actin2-R	**GACCACTAGTT**TT GTTTGTGTTTTGTGTTTTAAAACCACGAGCAAG	
Actin5-F	**CGCGGATCC**CAACAGGGAGTGGCGAAATGG	
Actin5-R	**CACGACGCGT**CTTGAAGTTTTATGATTTTTCAC TACAAAGACAGTAGATACG	
ActinA3a-F	**GGAAGATCT**CACGGTCGCTGCTATTTGTAACAAAAG	
ActinA3a-R	**CACGACGCGT**TTTGTTTTCTGAAAACAAAAAAAA AAATTATTAGTACTGAAATACACAC	
ActinA3a1-F	**GGAAGATCT**GCCAAACTTGTACGCATTGCCC	
ActinA3a2-F	**GGAAGATCT**CATCCCCACAATGGGCTATAACAG	
ActinA3a3-F	**GGAAGATCT**GTCTCTACTGATAAGGAGCCACTGG	
Pub-F	**GGAAGATCT**GTAAATACAACTCCAGACACCCAT	
Pub-R	**CGACGCGT**TTTGGAAAGGG TGCTACAATAACG	
Pub1-F	**GGAAGATCT**GTCGGAAATGCTGAAATGGCG	
Pub2-F	**GGAAGATCT**CGATCACATGCAATTGCAACGTGAG	
SPLNK#1	CGAAGAGTAACCGTTGCTAGGAGAGACC	Identify insertion sites
SPLNK#2	GTGGCTGAATGAGACTGGTGTCGAC	
5′SPLNK-PB#1	ACCGCATTGACAAGCACG	
5′SPLNK-PB#2	CTCCAAGCGGCGACTGAG	
15-F	CAACGGCAGTGAGGGCTGATTAGTGAAGAGC	
15-R	GCACAGCTTTCAGTAATTCGCACTCCTTGCGC	
pcr-mScarlet-F	CCGTGGTGGAACAGTACGAGC	RT-qPCR
qRT-SV40-R	CCTCTACAAATGTGGTATGGCTG	
qpcr-αtub-F	CCATCCATGTCGGTCAAGCTG	
qpcr-αtub-R	CATCTGGCCATCAGGCTGGATAC	

## Data Availability

The original contributions presented in this study are included in the article/Supplementary Material. Further inquiries can be directed to the corresponding authors.

## Supplementary Materials

The following supporting information can be downloaded at: https://www.mdpi.com/article/10.3390/insects17030349/s1, Figure S1: Schematic diagram of the *BdActA3a* upstream sequence; Table S1: Detailed information of the *BdActin* and *BdPUb* promoters; Table S2: Plasmids and their GenBank Accession Numbers; Table S3: Microinjection and transient expression of different *BdActin* promoter plasmids; Table S4: Microinjection and transient expression of *BdAcA3a* and *BdPUb* promoter plasmids with different lengths; Table S5: Microinjection for fluorescent strain construction; Table S6: Number of fluorescent G1 individuals obtained from the five positive cages.
