# Supplementary material for: Assessment of Ubiquitous Promoters Driving Fluorescent Marker and Transposase Expression to Develop a High-Performance piggyBac Transgenic System in Bactrocera dorsalis
Source: Insects. 2026 Mar 23;17(3):349. doi: 10.3390/insects17030349 (PMC13026108; doi:10.3390/insects17030349)
Supplement: Supplementary file 1 [file insects-17-00349-s001.zip › Table S1.pdf]

Table S1 Detailed information of the *BdActin* and *BdPUb* promoters.

Promoter name	Gene ID	Location	Enzyme(5'/3')
BdAct2	LOC105225453	Chromosome 3 - NC_064305.1<br>89,901,486..89,903,446	*BamH*I/*Spe*I
BdAct5	LOC105228222	Chromosome 4 - NC_064306.1<br>73,531,858..73,533,548	*BamH*I/*Mlu*I
BdActA3a-5.0 kb	LOC105222768	NW_026038098.1<br>17,324..22,282	*Bgl*II/*Mlu*I
BdActA3a-4.3 kb	LOC105222768	NW_026038098.1<br>17,933..22,282	*Bgl*II/*Mlu*I
BdActA3a-3.6 kb	LOC105222768	NW_026038098.1<br>18,660..22,282	*Bgl*II/*Mlu*I
BdActA3a-3.2 kb	LOC105222768	NW_026038098.1<br>19,102..22,282	*Bgl*II/*Mlu*I
BdPUb-3.6 kb	LOC105226185	Chromosome 4 - NC_064306.1<br>2,635,706..2,639,275	*Bgl*II/*Mlu*I
BdPUb-2.5 kb	LOC105226185	Chromosome 4 - NC_064306.1<br>2,636,719..2,639,275	*Bgl*II/*Mlu*I
BdPUb-1.6 kb	LOC105226185	Chromosome 4 - NC_064306.1<br>2,637,638..2,639,275	*Bgl*II/*Mlu*I
