# Supplementary material for: Assessment of Ubiquitous Promoters Driving Fluorescent Marker and Transposase Expression to Develop a High-Performance piggyBac Transgenic System in Bactrocera dorsalis
Source: Insects. 2026 Mar 23;17(3):349. doi: 10.3390/insects17030349 (PMC13026108; doi:10.3390/insects17030349)
Supplement: Supplementary file 1 [file insects-17-00349-s001.zip › Table S2.pdf]

Table S2 Plasmids and their GenBank Accession Numbers.

Plasmid	GenBank accession number
BdAct5>mScarlet-I	PZ124496
BdAct2>mScarlet-I	PZ124497
BdActA3a-5.0 kb>mScarlet-I	PZ124499
BdPub-3.6 kb>mScarlet-I	PZ124498
BdPUb-3.6 kb>hyPBase	PZ124500
