# Supplementary material for: Assessment of Ubiquitous Promoters Driving Fluorescent Marker and Transposase Expression to Develop a High-Performance piggyBac Transgenic System in Bactrocera dorsalis
Source: Insects. 2026 Mar 23;17(3):349. doi: 10.3390/insects17030349 (PMC13026108; doi:10.3390/insects17030349)
Supplement: Supplementary file 1 [file insects-17-00349-s001.zip › Table S3.pdf]

Table S3 Microinjection and transient expression of different *BdActin* promoter plasmids.

Plasmids	Injected embryos	Hatched larvae (hatching rate)
BdAct2>mScarlet-I	92	47（51.08%）
BdAct5>mScarlet-I	105	62（59.05%）
BdActA3a-3.2 kb>mScarlet-I	186	109（58.60%）
ddH_2O	100	57（57.00%）
