# Supplementary material for: Assessment of Ubiquitous Promoters Driving Fluorescent Marker and Transposase Expression to Develop a High-Performance piggyBac Transgenic System in Bactrocera dorsalis
Source: Insects. 2026 Mar 23;17(3):349. doi: 10.3390/insects17030349 (PMC13026108; doi:10.3390/insects17030349)
Supplement: Supplementary file 1 [file insects-17-00349-s001.zip › Table S4.pdf]

Table S4 Microinjection and transient expression of *BdAcA3a* and *BdPUb* promoter plasmids with different lengths.

**Plasmids**	**Injected embryos**	**Hatched larvae (hatching rate)**
BdActA3a-5 kb>mScarlet-I	169	113（66.86%）
BdActA3a-4.3 kb>mScarlet-I	173	89（51.45%）
BdActA3a-3.6 kb>mScarlet-I	166	122（73.49%）
BdActA3a-3.2 kb>mScarlet-I	157	97（61.78%）
BdPUb-3.6 kb>mScarlet-I	175	102（58.29%）
BdPUb-2.5 kb>mScarlet-I	128	79（61.72%）
BdPUb-1.6 kb>mScarlet-I	170	87（51.18%）
ddH$_2$O	86	51（59.30%）
