# Supplementary material for: Assessment of Ubiquitous Promoters Driving Fluorescent Marker and Transposase Expression to Develop a High-Performance piggyBac Transgenic System in Bactrocera dorsalis
Source: Insects. 2026 Mar 23;17(3):349. doi: 10.3390/insects17030349 (PMC13026108; doi:10.3390/insects17030349)
Supplement: Supplementary file 1 [file insects-17-00349-s001.zip › Table S5.pdf]

Table S5 Microinjection for fluorescent strain construction.

**Injection solution**	**Injected embryos**	**Hatched larvae (hatching rate)**	**Fluorescent hatched larvae**	**Fluorescent pupae**	**Fluorescent adults**
pPPUbMS＋BdPUb:hyPBase	1329	768（57.79%）	140	86	63
