# Supplementary material for: Assessment of Ubiquitous Promoters Driving Fluorescent Marker and Transposase Expression to Develop a High-Performance piggyBac Transgenic System in Bactrocera dorsalis
Source: Insects. 2026 Mar 23;17(3):349. doi: 10.3390/insects17030349 (PMC13026108; doi:10.3390/insects17030349)
Supplement: Supplementary file 1 [file insects-17-00349-s001.zip › Table S6.pdf]

**Table S6** Number of fluorescent G1 individuals obtained from the five positive cages.

| | Embryos | Pupae | Adults |
|---|---|---|---|
| No. 1 | 108 | 76 | 71 |
| No. 5 | 121 | 89 | 84 |
| No. 6 | 119 | 82 | 78 |
| No. 13 | 107 | 69 | 68 |
| No. 15 | 128 | 77 | 74 |
