# Supplementary figures and images for: Assessment of Ubiquitous Promoters Driving Fluorescent Marker and Transposase Expression to Develop a High-Performance piggyBac Transgenic System in Bactrocera dorsalis
Source: Insects. 2026 Mar 23;17(3):349. doi: 10.3390/insects17030349 (PMC13026108; doi:10.3390/insects17030349)

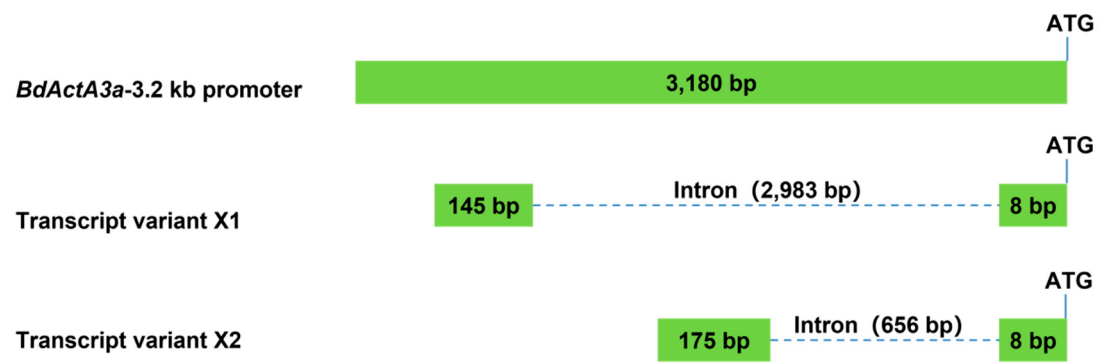

**Figure S1** Schematic diagram of the *BdActA3a* upstream sequence.

Supplement: Supplementary file 1 [file insects-17-00349-s001.zip › FIgure S1.pdf]
